# Notes and News

**Published:** 1889-10-05

**Authors:** 


### Notes and News.

Collected and Communicated.

We are glad to have it stated on authority that the Hospital Sunday Fund has now reached £41,600, and is just £1,000 in excess of the collections made in any previous year. The fund for 1889 closes on October 31.

The American Public Health Association, to which we refer elsewhere, will hold its seventeenth annual meeting in the Hall of the Brooklyn Institute, Washington and Concord States, on the four successive days, beginning on the 22nd of October and ending on the 25th.

There has been considerable excitement at Montreal about a patient who died under ether at the General Hospital. Dr. Kirkpatrick, the medical superintendent, refuses the name of the operator and anæsthetist, and Coroner Jones refuses to hold an inquest. It strikes us that this is a wrong principle; full information should always be supplied to the public, even though they take up a cry without sufficient cause.

The result of the West-end fever scare is that it was resolved at a meeting of the Committee of Works of St. George's, Hanover-square, to hold a special meeting, to consider with Prof. Corfield, the medical officer of health, the provisions of the Infectious Diseases Act, and the best means to enforce notification of cases of infectious disease occurring within the parish. It was also decided to instruct Mr. Livingstone, the surveyor, to confer with the authorities of surrounding parishes, with a view to fixing a uniform rate chargeable on advertisement hoardings, in accordance with an Act recently passed, empowering vestries to deal with the subject.

*Blackwood's Magazine* has had to "ca' canny" this month anent the sensation it started about the lepers of Robben Island. The Colonial Secretary at Cape Town lately stated that in despite of defective management on the part of the local medical overseer, which formerly existed, the accommodation provided for the lepers on Robben Island was vastly superior to what they could obtain in their own homes. The retention of the patients on the island was voluntary on their part, and their relations were continually permitted to visit them. The Colonial Secretary further declared that measures for effecting further improvements n the treatment of lepers and for their compulsory segraion under the Leprosy Represssion Act, which would involve serious difficulties and expense, had engaged the attention of the Government for a long time past.

A courageous doctor has refused to be swindled. Last week Dr. J. W. Cook brought an action in the Edmonton county court to recover fees for attending a boy who had been run over, and whose father, an innkeeper, declined to pay, on the ground that the doctor was called in by the cabman who was the cause of the accident. His honour, Judge Abdy, characterised the defence as a very shabby one, and ordered the father of the boy to pay the amount claimed with costs.

At the rate at which we now live, and which tells so terribly on the nerves, there can be no doubt that the unceasing noises of town life must affect our health. We have become used to these noises, and can scarcely conceive what a relief and pleasure it would be to simply have all vehicles running on rubber tyres. Dr. Andrew Wilson has been calling attention to this subject, and denouncing the many needless noises which ruin the health, temper, and spirits of many modern men.

There is another thing to which we are accustomed, and yet which will surely strike our descendants as a relic of barbarism—our butchers' shops. We can quite imagine a philosopher of the next century studying a print showing a shop where carcases of sheep and bullocks hang in all their ghastly hideousness, and saying, "No wonder they had Jack-the-Rippers in those days, when such savage sights were to be seen in every public street." Certainly, many of us would enjoy our meals more if we less frequently encountered the sight and smell of our food before it was cooked.

Harvest Thanksgivings are now being held throughout the length and breadth of our land, and our hospitals are greatly benefitted thereby. Great baskets of fruit, vegetables, and flowers which have been used in decoration find their way to poor patients, to whom change of diet and the sight of flowers are cheering and invigorating. The mediæval idea of offering of the first-fruits of all the earth to the Lord is best carried out by thus displaying the offerings in the church and then forwarding them to the sick, for what is given to the least of these our brethren, is given unto the Lord.

Dr. Wilson gave voice to some very revolutionary sentiments at the Sanitary Congress. He said he doubted whether public opinion was sufficiently advanced to enforce certificates of health on the part of persons about to marry, but he thought the time was at hand to put some check on the appalling waste of infant life which disgraced our civilisation, by prohibiting any man to marry unless he could produce reasonable proof that he was in a position to maintain a wife. So long as the family medical practitioner continued to be paid to attend only on people when they were ill, and not to conserve the health of the household, there would be a constant drag on public health progress.

Prizes have been awarded at the Paris Exhibition, Social Economy Section, to the following associations: Association for the Oral Instruction of the Deaf and Dumb; the Chief Registrar of Friendly Societies; City and Guilds of London Institute; Commissioners of Sewers, London; Corporation of City of London; Foresters Affiliated Order; Mrs. Ernest Hart's Donegal Industrial Fund; Sir Sidney Waterlow's Improved Industrial Dwellings Company; Industrial Training Ship "Clio"; International Arbitration and Peace Association; Liberty and Property Defence League; Mansion House Council; Metropolitan Asylums Board; Metropolitan Public Gardens Association; People's Palace; Reformatory and Industrial Schools, London; School Board for London; St. John's Ambulance Association; Taylor Sedley; Working Men's Club and Institute.

The following vacancies are announced this week, the amount of the salary in each case being given after the name of the institution :

**Resident Medical Officers.**

Jersey Dispensary and Infirmary. Medical Officer.—£120, rooms, &c.
Brompton Consumption Hospital. House Physician.
Gateshead Dispensary. House Surgeon.—£200, rooms, &c.
Paddington Green Children's Hospital. House Surgeon.—£50, board, &c.

**Non-Resident Medical Officers.**

Western General Dispensary. Surgeon Dentist.
Dundee University College. Professor of Physiology.—£350.
Western General Dispensary. Honorary Surgeon.
University of Sydney. Professor of Anatomy.
Plymouth Public Dispensary. Assistant Physician.—£60.
Ballinrobe Union. Medical Officer.—£100.

An enquiry was held at Shoreditch Infirmary regarding the death of an unknown man from starvation. The man was brought to the infirmary and refused admission by the portress. We should like to know who gave the portress authority to grant or refuse admission to patients ?

On September 25 Lord Londonderry performed the formal opening of the extension of the Hartlepools' Hospital, which owes its original erection largely to the munificent support of the Burdon family, and which it has now been found needful to enlarge to meet the growing needs of the Hartlepools and their increasing population. The cost of the new portion has been £4,000. The designs were by Mr. W. Lister Newcombe.

It may be very poetical to wail the summer fled and the approach of ice and snow, but apparently the winter has its compensations. The report of Commissioners to Scottish Lunatic Asylums, just published, distinctly proves that during the summer the admissions to asylums nearly double those of winter. Dr. A. Mitchell has also proved that suicide is much more frequent in the summer months ; and all asylum attendants agree that patients are more refractory during the long hot days. If, therefore, the summer induces insanity and suicide we can part with its sunny days with less regret.

Birmingham General Hospital, being in want of funds, the mayor has been speaking strongly on the subject. He said that the idea seemed to have prevailed that whatever other charity suffered the General Hospital would not do so, but this idea was incorrect, because at the present time the General Hospital required more assistance than any other charity in Birmingham. It had a debt of £9,000, and its subscription list, instead of increasing, was actually decreasing. Since the last Hospital Sunday collection in aid of its funds the annual subscription list had fallen off £360. Decreasing funds and increasing patients have at last brought matters to a crisis, hence the present appeal for aid.

An extraordinary demonstration of hypnotism was given last Saturday by the Rev. Arthur Tooth at Woodside Orphanage. Several boys were thrown into a hypnotic sleep and put through strange antics. It is interesting to note that Mr. Tooth has now many patients whom he treats, and, according to his account, with great success, for dipsomania. Of these, a few ladies are resident at his house, others call at regular intervals to see him. In describing a case of infantile paralysis, which had greatly improved under his treatment, and the patient concerned in which he produced, Mr. Tooth was asked by a medical gentleman whether he claimed to be able to reconstruct the nerve cells, which in such cases became destroyed. Mr. Tooth, in effect, replied that nature reconstructed the cells upon his appeal. The medical man rejoined that that was a creation which it was difficult to follow. Several doctors were amongst the audience.

Dr. Jeaffreson, of Newcastle-on-Tyne, has compiled a "Nursing Chart," which offers an easy and simple method of recording what a patient has taken in the way of medicine, food, stimulants, etc., and the effects of these upon the system generally, and the secretions. Each sheet of the chart represents twelve hours nursing, and thirty of them, neatly printed and ruled, are mounted on a stout board with printed illustrations at the back.

The present Sandgate Convalescent Home is about to be enlarged. Attached to Beach Rocks there is an ample plot of ground now laid out as a lawn. The new building w ll be erected on it (in conjunction with that which exists and will be rebuilt), and will provide a home for 156 patients The estimated cost is about £10,000, and an additional sum of about £1,500 will be needed in refurnishing the same. t is proposed that "In Memoriam" rooms should be provided as appropriate remembrancers of departed friends, or in token of gratitude for special blessings. Donors to the building fund can have rooms named either after thei deceased friends or themselves.

One of the most serious lessons taught by the medical faculty is the law of heredity, the doctrine that the sins of the fathers descend to the children. Dr. Brown-Sequard long ago proved that by artificially inducing epilepsy in guinea-pigs the disease was transmitted to the offspring ; and this is a clear example of the possibility of engendering conditions in living beings which are calculated to affect their offspring and to modify their descendants. We have all known cases in which consumption, scrofula, and insanity, have reappeared in the progeny of those affected ; and this view cannot be kept too clearly before the public. The *Scotsman* has lately had an article on "Disease and Evolution," treating this subject, and plainly setting forth how our vices not only injure ourselves, but the whole human race.

An interesting table, showing the assigned causes of insanity in the cases of all patients admitted into public and private asylums in England and Wales during the ten years 1878-87, is given in the report of the Commissioners in Lunacy just issued. These causes are not taken from the statements in the papers of admission of the patients, but are those which have been verified by the medical officers of the asylums. The total number of admissions during the ten years was 136,478, being 66,918 of the male, and 69,560 of the female sex. Amongst the causes enumerated are the following: Domestic trouble (including loss of relatives and friends), males 2,787, females 6,782 ; adverse circumstances (including business anxieties and pecuniary difficulties), m. 5,493, f. 2,567 ; mental anxiety and "worry" (not included under the above two heads), and overwork, m. 4,435, f. 3,843 ; religious excitement, m. 1,693, f. 2,076 ; love affairs, etc., m. 456, f. 1,768 ; fright and nervous shock, m. 639, f. 1,314 ; intemperance in drink, m. 13,286, f. 5,004; over-exertion, m. 449, f. 312; sunstoke, m. 1,557, f 129 ; accident or injury, m. 3,497, f. 702; fevers, m. 489, f. 391; privation and starvation, m. 1,112; f. 1,495; old age, m. 2,568, f. 3,205; other bodily diseases or disorders, m. 7,420, f. 7,299 ; previous attacks, m. 9,656, 13,138 hereditary influence ascertained, m. 12,703, f. 15,360 ; con genital defect ascertained, m. 3,461, f. 2,420 ; other ascertained causes, m. 1,584, f. 738 ; unknown, m. 14,286, f. 13,985. The total number of lunatics, idiots, and person of unsound mind in England and Wales on January 1 last was 84,340, being an increase of 1,697 on the figures of the previous year. The ratio to the whole population has risen from 28·87 to 29·07 per 10,000, which is the highest point at which it has stood. The rate of recovery to the admissions calculated at 38·71 per cent.

